# Supplementary material for: Improving the Maternity Care Safety Net: Establishing Maternal Mortality Surveillance for Non-Obstetric Providers and Institutions
Source: Int J Environ Res Public Health. 2023 Dec 27;21(1):37. doi: 10.3390/ijerph21010037 (PMC10815856; doi:10.3390/ijerph21010037)
Supplement: Supplementary file 1 [file ijerph-21-00037-s001.zip › ijerph-2665619-supplementary.pdf]

Case Identification Codes

Delivery DX, PR, DRGs	
**ICD-9-CM**	**ICD-10-CM/PCS**
Any DX of V27	Any DX of Z37X: Z370, Z371, Z372, Z373, Z374, Z3750, Z3751, Z3752, Z3753, Z3754, Z3759, Z3760, Z3761, Z3762, Z3763, Z3764, Z3769, Z377, Z379
Any DX of 650 (normal delivery)	Any DX of O80 (vaginal delivery)
	Any DX of O82
Any DX of '66970', '66971' (C-section)	
Any DRG:<br>DRG 765-768 or 774-775 if DRGVER >= 25 (after Q4 2007) and<br>DRG 370-375 if DRGVER < 25 (before Q4 2007)	DRG depending on the version:<br>DRG 765-768 or 774-775 if DRGVER >= 25 (after Q4 2007) and<br>DRG 370-375 if DRGVER < 25 (before Q4 2007)
Prior to Oct 2007; DRG version < 25<br>370 = '370: CESAREAN SECTION W CC'<br>371 = '371: CESAREAN SECTION W/O CC'<br>372 = '372: VAGINAL DELIVERY W COMPLICATING DIAGNOSES'<br>373 = '373: VAGINAL DELIVERY W/O COMPLICATING DIAGNOSES'<br>374 = '374: VAGINAL DELIVERY W STERILIZATION &/OR D&C'<br>375 = '375: VAGINAL DELIVERY W O.R. PROC EXCEPT STERIL &/OR D&C'	Starting FY2016 (Oct 2015); MS-DRG version 33-35<br>765 = '765: CESAREAN SECTION W CC/MCC'<br>766 = '766: CESAREAN SECTION W/O CC/MCC'<br>767 = '767: VAGINAL DELIVERY W

	STERILIZATION &/OR D&C'<br>768 = '768: VAGINAL DELIVERY W O.R. PROC EXCEPT STERIL &/OR D&C'<br>774 = '774: VAGINAL DELIVERY W COMPLICATING DIAGNOSES'<br>775 = '775: VAGINAL DELIVERY W/O COMPLICATING DIAGNOSES'
<u>Starting FY2008 (Oct 2007); MS-DRG versions 25-32</u><br>765 = '765: CESAREAN SECTION W CC/MCC'<br>766 = '766: CESAREAN SECTION W/O CC/MCC'<br>767 = '767: VAGINAL DELIVERY W STERILIZATION &/OR D&C'<br>768 = '768: VAGINAL DELIVERY W O.R. PROC EXCEPT STERIL &/OR D&C'<br>774 = '774: VAGINAL DELIVERY W COMPLICATING DIAGNOSES'<br>775 = '775: VAGINAL DELIVERY W/O COMPLICATING DIAGNOSES'	<u>Starting FY 2019 (Oct 2018); MS-DRG version >= 36</u><br>MS-DRG 768 Vaginal Delivery with O.R. procedure except Sterilization &/or D&C<br>MS-DRG 783 Cesarean Section with Sterilization with MCC<br>MS-DRG 784 Cesarean Section with Sterilization with CC<br>MS-DRG 785 Cesarean Section with Sterilization without CC/MCC<br>MS-DRG 786 Cesarean Section without Sterilization with MCC<br>MS-DRG 787 Cesarean Section without Sterilization with CC

	MS-DRG 788 Cesarean Section without Sterilization without CC/MCC<br>MS-DRG 796 Vaginal Delivery with Sterilization/D&C with MCC<br>MS-DRG 797 Vaginal Delivery with Sterilization/D&C with CC<br>MS-DRG 798 Vaginal Delivery with Sterilization/D&C without CC/MCC<br>MS-DRG 805 Vaginal Delivery without Sterilization/D&C with MCC<br>MS-DRG 806 Vaginal Delivery without Sterilization/D&C with CC<br>MS-DRG 807 Vaginal Delivery without Sterilization/D&C without CC/MCC
Any procedures codes '720','721','7221','7229','7231','7239','724','7251','7252','7253','7254','726',' 7271', '7279','728','729','7322','7359','736' ,'740' ,'741','742' ,'744' ,'7499'	Any procedure codes 10D00Z0-10D00Z2, 10D07Z3-0D07Z8, 10E0XZZ
Any DX of 630-639	Any DX of 'O00', 'O01', 'O02', 'O03', 'O04', 'O07', 'O08' or ICD10_PR=10A0XX

Any Procedures codes '6901','6951','7491',750'	PR starting with '10D0' and 10E0XZZ.
**C-Sections DX, PR, and DRGs**	
Any DX of '66970', '66971'	
Any PR '740','741','742','744','7499'	
Any DRG:<br>MS-DRGs 765-766 (DRG version is 25-32)<br>DRGs 370-371 (DRG version < 25)<br>MS-DRGs 765-766 (DRG version is 33-35)<br>MS-DRGs 783-787 (DRG version >= 36)	
